# SMRT Sequencing Revealed Mitogenome Characteristics and Mitogenome-Wide DNA Modification Pattern in *Ophiocordyceps sinensis*

**DOI:** 10.3389/fmicb.2017.01422

**Published:** 2017-07-27

**Authors:** Xincong Kang, Liqin Hu, Pengyuan Shen, Rui Li, Dongbo Liu

**Affiliations:** ^1^Hunan Provincial Key Laboratory of Crop Germplasm Innovation and Utilization, Hunan Agricultural University Changsha, China; ^2^Horticulture and Landscape College, Hunan Agricultural University Changsha, China; ^3^State Key Laboratory of Subhealth Intervention Technology Changsha, China; ^4^Nextomics Biosciences Wuhan, China; ^5^Hunan Co-Innovation Center for Utilization of Botanical Functional Ingredients Changsha, China

**Keywords:** *Ophiocordyceps sinensis*, mitochondrial genome, characteristics, mitochondrial epigenetics, methylation, *rps3*

## Abstract

Single molecule, real-time (SMRT) sequencing was used to characterize mitochondrial (mt) genome of *Ophiocordyceps sinensis* and to analyze the mt genome-wide pattern of epigenetic DNA modification. The complete mt genome of *O. sinensis*, with a size of 157,539 bp, is the fourth largest Ascomycota mt genome sequenced to date. It contained 14 conserved protein-coding genes (PCGs), 1 intronic protein *rps3*, 27 tRNAs and 2 rRNA subunits, which are common characteristics of the known mt genomes in Hypocreales. A phylogenetic tree inferred from 14 PCGs in Pezizomycotina fungi supports *O. sinensis* as most closely related to *Hirsutella rhossiliensis* in Ophiocordycipitaceae. A total of 36 sequence sites in *rps3* were under positive selection, with dN/dS >1 in the 20 compared fungi. Among them, 16 sites were statistically significant. In addition, the mt genome-wide base modification pattern of *O. sinensis* was determined in this study, especially DNA methylation. The methylations were located in coding and uncoding regions of mt PCGs in *O. sinensis*, and might be closely related to the expression of PCGs or the binding affinity of transcription factor A to mtDNA. Consequently, these methylations may affect the enzymatic activity of oxidative phosphorylation and then the mt respiratory rate; or they may influence mt biogenesis. Therefore, methylations in the mitogenome of *O. sinensis* might be a genetic feature to adapt to the cold and low PO_2_ environment at high altitude, where *O. sinensis* is endemic. This is the first report on epigenetic modifications in a fungal mt genome.

## Introduction

*Ophiocordyceps sinensis* (syn. *Cordyceps sinensis*) is an entomopathogenic fungus that infects larvae of Hepialidae ghost moths to form a parasitic complex called “DongChongXiaCao” in Chinese (Commission, 2015). “DongChongXiaCao” is a Traditional Chinese Medicine treatment that has been used for 1000s of years for respiratory, renal, liver and cardiovascular diseases, hyposexuality and hyperlipidemia (Zhu et al., 1998; Yue et al., 2013). *O. sinensis* is endemic to alpine regions on the Tibetan Plateau, with 3000 m as the lowest altitude for the distribution (Li et al., 2011). This fungus is rare in natural resources because of its strict host-specificity, limited geographical distribution and over-exploitation in recent decades (Li et al., 2011).

The mitochondrion is a cellular organelle that is required for respiratory metabolism and ATP production. In the mitochondrion, ATP synthesis is catalyzed by five mitochondrial inner membrane-bound enzyme complexes (Complexes I–V) (Saraste, 1999; Chandel and Schumacker, 2000). Complexes I–V are NADH-ubiquinol oxidoreductase, succinate-ubiquinol oxidoreductase, ubiquinol-cytochrome C oxidoreductase, cytochrome C oxidase, and ATP synthase, respectively. The mt genome encodes for seven subunits of Complex I (NAD1-6, NAD4L), one subunit of Complex III (COB), three subunits of Complex IV (COX1-3), and three subunits of Complex V (ATP6, ATP8, and ATP9) (Saraste, 1999). In addition to the genes encoding OXPHOS proteins, two rRNA subunits and a set of tRNA genes are included in the fungal mt genome. Gene content is highly conserved in mitochondria of various fungi, but some characteristics, such as gene order, tRNA gene clusters, intergenic regions, mobile elements and introns, can be highly variable (Ghikas et al., 2006; Aguileta et al., 2014; Losada et al., 2014; Mardanov et al., 2014). As of December 2016, there were 179 Ascomycota mt genomes submitted to GenBank, and *Sclerotinia borealis* (NC_025200, 203,051 bp) had the largest mt genome (Mardanov et al., 2014)^1^. Due to its small size, fast evolution, high copy number, and relatively conserved gene content, the mt genome has been successfully used in evolutionary biology and phylogeny (Joardar et al., 2012).

The present understanding of the mt epigenome has gone through a series of evolutions, and conflicting data about epigenomes existed in the 1970s and early 1980s due to the uneven distribution of methylation patterns and low sensitivity of detection methods (Castegna et al., 2015). A few studies have shown mt methylation while others have reported the absence of any methylation in the mitochondrion (Cummings et al., 1974; Dawid, 1974; Groot and Kroon, 1979; Pollack et al., 1984). The advent of next generation sequencing technologies has provided opportunities for characterizing mt genome-wide cytosine methylation. Ghosh et al. (2014) reported the first genome-wide map of human mitochondrial methylation. Ghosh et al. (2016) revealed that it was dynamic in nature of the hydroxymethly cytosine marking in the human mitochondrial genome. However, little trace of DNA modification on mtDNA is detected in fungi. The current methodologies for epigenomics, based on bisulfite, enrichment, or sequencing and PCR, impede the processing of the epigenetic study due to the limits in the resolution, modification type, locus and cost (Mensaert et al., 2014).

Single molecule, real-time sequencing has created significant progress in read length and sequencing of base modification sites (Flusberg et al., 2010; Feng et al., 2013). SMRT sequencing can produce read lengths of up to 40 kb and direct sequencing of base modification. The most important characteristics of SMRT sequencing are that SMRT sequencing (1) could be free from amplification biases and (2) easily sequenced the complex regions containing secondary structure in low coverage (Koren et al., 2013). DNA modifications across the genome could be directly determined by the kinetics of DNA synthesis, because SMRT sequencing monitors the processing of single DNA molecules by DNA polymerase (Eid et al., 2009; Feng et al., 2013). The biggest concern in SMRT sequencing is the relatively high error rate, which can be effectively decreased through multiple sequencing passes. Long reads and a circular consensus sequencing strategy make SMRT convenient for: *de novo* assembly of mitochondria, chloroplasts, and microbial genomes, especially for complex genomes; characterizing genomic structural variations; and analyzing targeted sequencing regions or modification sites (Carneiro et al., 2012; Grad et al., 2012; Chin et al., 2013; Feng et al., 2013; Koren et al., 2013).

In the current study, we sequenced the mt genome of wild fungus *O. sinensis* using SMRT sequencing technology on a PacBio RS II sequencing platform. We described the gene content and genomic organization of this high-altitude fungus, and performed a comparative analysis with the sequenced Hypocreales mt genomes. The main focus in this study is on the mitogenome-wide epigenetic DNA modification pattern of *O. sinensis*. This is the first report on epigenetic modifications in a fungal mt genome, and it will provide the basis for further research on mt epigenetics of Hypocrealean fungi.

## Materials and Methods

### Sample Collection

Sample of the fungus *O. sinensis* (CCTCC AF 2017003) was from the fruiting body of the wild *O. sinensis*. Fresh specimens were purchased in a local market in Guoluo of Qinghai Province, China (Latitude 34.48°N, Longitude 100.23°E). Governmental permission is not required for *O. sinensis* purchases in local markets, and the collections of *O. sinensis* specimen sold by local farmers fall under the governmental regulations for traditional Chinese herbal products. All fresh *O. sinensis* specimens were washed thoroughly on site in running water with gentle brushing, soaked in 75% ethanol for 10 min for surface sterilization and washed again three times with sterile water. These samples were snap-frozen in liquid nitrogen immediately after sampling, transported to our laboratory by using the solid carbon dioxide, and stored at -80°C until further use. The wild *O. sinensis* was preliminarily identified according to the morphological characteristics and several nuclear loci, including internal transcribed spacer region (ITS), small and large 18S nuclear ribosomal RNA subunits (nrSSU, nrLSU) and translation elongation factor 1-alpha (TEF-1α). These four genes (GenBank accession number: MF403011, MF403012, MF403013, MF425658) all showed the highest homology to *O. sinensis* ().

### DNA Extraction and Genome Sequencing

Total DNA was extracted from the stroma of *O. sinensis* by a modified CTAB method as previously described (Kang et al., 2011). The integrity, quality and concentration of total DNA were analyzed by agarose gel electrophoresis, NanoDrop 1000 spectrophotometer and Qubit fluorometer. DNA was randomly sheared to fragments with an average size of 20 kb by using g-TUBE. Sheared DNA was then DNA damage repaired and end repaired. SMRTbell templates were obtained by ligating the blunt hairpin adapters to the ends of the repaired fragments, followed by the addition of exonuclease to remove failed ligation products. Before annealing the sequencing primer and binding polymerase to SMRTbell templates, the quality of library was assessed by an Agilent 2100 Bioanalyzer High Sensitivity Kit. Eight SMRT cells were sequenced using P6-C4 reagents on a PacBio RS II sequencing platform (Pacific Biosciences, Nextomics Biosciences, Co., Ltd, Wuhan).

### Assembly of the Mitochondrial Genome

Clean data were obtained by filtering out the sequencing adapters and low-quality sequences (parameters: minimum sub-read length = 500 bp; minimum polymerase read quality = 0.80). The mt sequences were extracted from the filtered reads containing both nuclear and mt genomes, using BLASR which matches each read against 201 published fungal mitochondrial genomes (Chaisson and Tesler, 2012). About 2815 subreads in 25.4 Mb mt sequencing data were obtained, with an average read length of 8993 bp and a longest read length of 39,358 bp, reaching an average depth of 167 X. The mt genome was assembled through Hierarchical Genome Assembly Process (HGAP) workflow, including preassembly, error correction, Celera assembly and polishing with Quiver (Chin et al., 2013). Long reads were selected to be “seed” reads, which the other subreads were blast against to improve accuracy. The corrected reads were retained and fully assembled by using overlap-layout-consensus (OLC) algorithm in the Celera Assembler program (Myers et al., 2000), and then further refined with Quiver (Chin et al., 2013).

### Mitochondrial Genome Annotation

Genes in *O. sinensis* mt genome were predicted by MFannot, RNAweasel^2^ and BLASTn against NCBI Organelle Genome Resources^3^ (Altschul et al., 1990; Lang et al., 2007; Valach et al., 2014). The PCGs and rRNA genes were identified by MFannot and checked by BLASTn against Hypocreales (Altschul et al., 1990), while the tRNA genes were checked using RNA weasel. Intron–exon boundaries of the PCGs were adjusted manually on the basis of BLASTn against multiple Hypocreales mt coding sequence (Altschul et al., 1990). The nucleotide sequences of PCGs were translated to protein sequences using the Mold, Protozoan and Coelenterate Mitochondrial code (transl_table = 4). Open reading frames (ORFs > 100 bp) in the intergenic and intronic regions were predicted by MFannot (Valach et al., 2014). Predicted ORFs were analyzed by InterProScan^4^. The mitochondrial genome map was generated with Circos software (Krzywinski et al., 2009) and CLC Sequence Viewer 7.8.1 (CLC bio Inc., Cambridge, MA, United States), and then modified by Adobe Illustrator CC 2015 (Version 19.0.0.44, Adobe, San Jose, CA, United States).

### Analysis of Repetitive Sequences

Local BLASTn search of mtDNA against itself was performed using a cut-off e-value of 10^-7^ (Altschul et al., 1990). Repetitive sequences were analyzed by several programs, including REPuter, Tandem Repeats Finder and MIcroSAtellite (Benson, 1999; Kurtz et al., 2001; Thiel et al., 2003). REPuter was applied to identify the forward, reverse, complement and palindromic sequences. Tandem Repeats Finder was used to find tandem repeats while MIcroSAtellite detected the microsatellite DNA (1–6 bp).

### Methylation Modification

Pacific Biosciences’ SMRTPortal analysis platform v. 1.3.1 was used to identify modified positions. At each genomic position, modQVs were computed as the -10 log (*P*-value) for representing a modified base position, based on the distributions of the kinetics of interpulse durations (IPD ratios) from all reads covering this position and from in silico kinetic reference values (Feng et al., 2013). A value of 20 is the minimum default threshold and corresponds to a *P*-value of 0.01. DNA methylation on both mtDNA strands was assessed independently and represented by modQV, which comprises base incorporation rates differing from that of the unmodified reference sequences. The RS_Modification_and_Motif_Analysis.1 protocol of the SMRT analysis v2.0 was used to identify methylation and the corresponding motifs of the responsible DNA methylases.

### Phylogenetic Analysis

A phylogenetic tree was inferred by the ML method using the nucleotides of 14 concatenated PCGs (*atp6, atp8, atp9, cox1-3, nad1-6, nad4L, cob*) in mt genome. The 20 fungal mt genomes downloaded from NCBI were shown in Supplementary Table S1. Multiple sequence alignment was performed using the MAFFT program (Katoh and Standley, 2013). Poorly aligned positions and gap positions were removed with Gblocks (Castresana, 2000). Three species belonging to Glomerellales (*Colletotrichum acutatum, Colletotrichum lupini*) and Eurotiales (*Penicillium polonicum*) were used as outgroup taxa in the phylogenetic analysis. The best model used in the ML phylogenetic tree was determined by using “find best DNA/protein models (ML)” in MEGA 7.0 (Kumar et al., 2016). The “GTR + G” model produced the lowest values for both the Bayesian Information Criterion and the corrected Akaike information criterion, therefore it was chosen for phylogenetic analysis. The phylogenetic tree was constructed using RAxML 8.0.19 with 500 bootstrap replicates (Stamatakis, 2006). BI analyses were processed with 1,000,000 generations and four chains (one cold and three hot chains), with sampling every 500 generations and a burn-in of 25% (Ronquist and Huelsenbeck, 2003). The confidence values of the BI tree were shown as Bayesian posterior probabilities in percentages. The NJ phylogenetic tree based on 14 PCGs or *rps3* was constructed by MEGA 7.0 and the evolutionary distances were computed using the Tajima-Nei method.

For *rps3* analysis, the outgroup *P. polonicum* was changed to *Penicillium nordicum* to avoid a large number of gaps and to get more genetic information. Because *rps3* gene in *P. polonicum* contained a premature termination codon, resulting a shorter aa sequence (Kang et al., 2016). The dN, dS, and the ratio (dN/dS) of each *rps3* sequence with the reference of *P. nordicum* were calculated using CODEML (RateAncestor = 1) in PAML v4.7a (Yang, 1997; Yang and Nielsen, 2000). Sequence alignment was performed for *rps3* genes in Hypocreales (length from 398 to 544 amino acids) using ClustalW version 2.0.12 (Sievers et al., 2011). The positive selection pressure on *rps3* was detected using CODEML implemented in PAML v4.8 (Yang and Nielsen, 2002; Yang et al., 2005; Yang, 2007; Lin et al., 2015). Excluding the ambiguous sequences, the *rps3* gene sequences were represented by 322 consensus aa sequences. The models of M0 (one-ratio), M1a (neutral), M2a (selection), M7 (beta), and M8 (β and ω) were used to calculate the dN/dS values.

## Results

### Characteristics of the *O. sinensis* mt Genome

**FIGURE 1:**
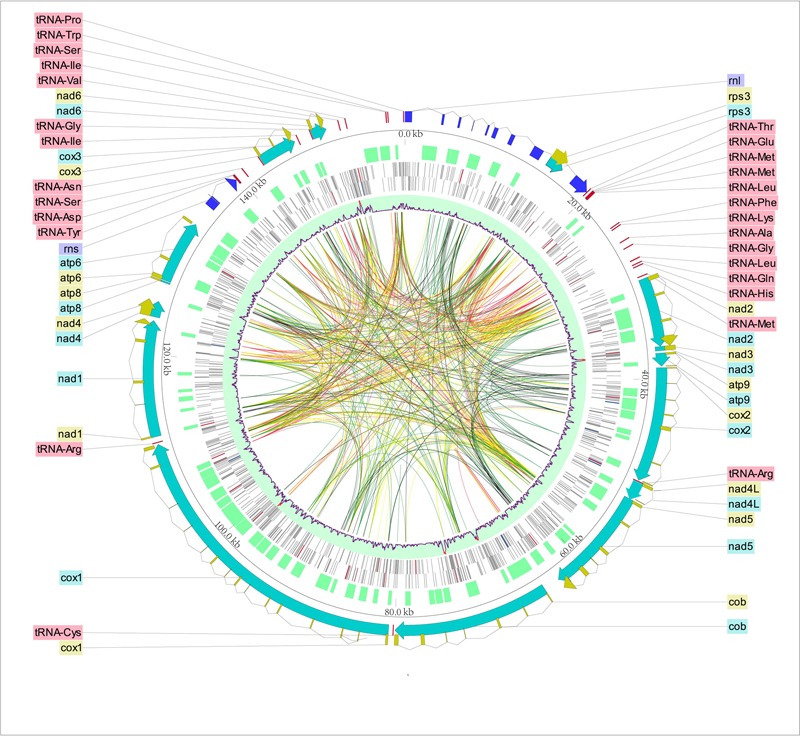
Circular map of the *Ophiocordyceps sinensis* mt genome. The scales indicate location in kb, starting with *rnl*. Using Circos and CLC Sequence Viewer, we integrated the gene annotation, DNA base modification, and some other information. From outermost to innermost: ring 1 with dingy yellow is CDS. Ring 2 includes protein-coding genes (lasureous), rRNAs (blue) and tRNAs (red). ORFs are located on ring 3. Rings 4 and 5 shows DNA base modifications sites in the forward and backward strand, respectively. Red lines indicate 4mC, blue lines show 6mA, while gray lines represent the other base modifications. Ring 6 with light blue as background color is the methylations for both strands in a 200 bp- sliding window. Red lines in the ring 6 mean that the number of the methylation sites in the sliding window is over 10. The ribbons inside the circle connect the repeat sequences with significant (e-value < 10^-7^) similarity (red: identity ≥ 90; yellow: 90 > identity ≥ 80; green: 80 > identity ≥ 70; gray: identity < 70).

The complete mt genome of *O. sinensis* was a circular double-stranded DNA molecule with 157,539 bp in length (**Table 1** and **Figure 1**, GenBank accession number: KY622006), which is similar to the *O. sinensis* mt genome (157,510 bp) reported by Li et al. (2015) and much larger than the other fungi in the same order Hypocreales (**Table 1**). The size variation of mt genome was caused by the length of intergenic regions, and the number, length of introns and accessory genes (**Table 1**) (Deng et al., 2016). Although the mtDNA size is in large variation (25,615–157,539 bp), the gene content slightly differed in Hypocreales (**Table 1**). There were 14 PCGs, 1 *rps3*, 27 tRNAs, and 2 rRNA subunits encoded in the mt genome. All protein and RNA coding genes were located on positive strand and orient clockwise (**Figure 1** and **Table 2**). In addition, there were 73 ORFs coding putative proteins in the *O. sinensis* mt genome (**Table 1** and Supplementary Table S2).

**Table 1:** Characteristics of mt genomes of Hypocreales fungi.

Species	Total length (bp)	AT (%)	Length (number) of PCGs (bp)	Length (number) of tRNAs (bp)	Length (number) of rRNAs (bp)	Intergenic region (bp)	Length (number) of introns	ORFs in introns	ORFs in intergenic regions
*Fusarium oxysporum*	34477	69.0	14484 (15)	2603 (25)	4742 (2)	11252	2738 (2)	2	0
*Acremonium chrysogenum*	27266	73.5	13305 (14)	1936 (26)	6236 (2)	4653	1330 (2)	0	3
*Metacordyceps chlamydosporia*	25615	71.7	14385 (15)	1625 (23)	4580 (2)	4691	1652 (1)	1	0
*Cordyceps militaris*	33277	72.2	14707 (15)	1924 (26)	4666 (2)	3863	9868 (8)	1	0
*Hypocrea jecorina*	42130	72.7	16149 (15)	1937 (26)	4369 (2)	10385	10933 (9)	1	4
*Hypomyces aurantius*	71638	71.7	16572 (16)	1870 (25)	4633 (2)	19416	30573 (17)	22	7
*Hirsutella minnesotensis*	52245	71.6	15171 (15)	1863 (25)	4898 (2)	14637	17123 (13)	12	4
*Hirsutella rhossiliensis*	62483	71.8	14571 (15)	1922 (26)	7067 (2)	28081	12477 (9)	7	3
*Tolypocladium ophioglossoides*	35159	72.4	14406 (15)	1869 (25)	4694 (2)	8230	7319 (6)	3	2
*Ophiocordyceps sinensis*	157539	69.8	14913 (15)	2010 (27)	6558 (2)	29350	106555 (54)	61	12

**Table 2:** Annotation of conserved PCGs and rRNA genes of *O. sinensis* mt genome.

Gene	Start	End	Length (bp)	Number of Introns	Intron length (bp)	Exon length (bp)	Coding sequence density	Start/stop codons	Strand
*rnl*	1	19712	19712	8	14805	4907	24.89%	-	+
*rps3*	14881	16464	1584	0	0	1584	100.00%	ATG,TAG	+
*nad2*	29906	36733	6828	3	5097	1731	25.35%	ATG,TAA	+
*nad3*	36734	37147	414	0	0	414	100.00%	ATG,TAA	+
*atp9*	37382	38676	1295	1	1070	225	17.37%	GTG,TAA	+
*cox2*	38810	50128	11319	6	10536	783	6.92%	ATG,TAA	+
*nad4L*	50363	52138	1776	1	1506	270	15.20%	ATG,TAA	+
*nad5*	52138	62723	10586	5	8591	1995	18.85%	ATG,TAA	+
*cob*	64342	79765	15424	6	14239	1185	7.68%	ATG,TAA	+
*cox1*	80387	111177	30791	14	29195	1596	5.18%	ATG,TAG	+
*nad1*	111941	123385	11445	4	10335	1110	10.04%	ATG,TAA	+
*nad4*	123751	125322	1572	0	0	1572	100.00%	ATG,TAA	+
*atp8*	127221	127367	147	0	0	147	100.00%	ATG,TAA	+
*atp6*	127453	133700	6248	2	5468	780	12.48%	ATG,TAA	+
*rns*	135867	139587	3721	1	2025	1696	45.58%	-	+
*cox3*	142715	146434	3720	2	2910	810	21.77%	GTG,TAG	+
*nad6*	148244	149731	1488	1	777	711	44.89%	ATG,TAA	+

In the *O. sinensis* mt genome, the 14 PCGs included 7 NADH dehydrogenases (*nad1*-*nad6, nad4L*), 3 cytochrome c oxidases (*cox1*-*cox3*), 3 ATP synthases (*atp6, atp8, atp9*), and 1 cytochrome b gene (*cob*), for encoding proteins involved in respiratory chain complexes. In addition, a *rps3* gene which encodes 40S ribosomal protein S3 was found in the intron of *rnl* (*rnl*_I8). The majority of the PCGs were split by several introns into multiple short exons (**Figure 1** and Supplementary Table S3). The introns (*n* = 54) covered ca. 67.64% of the whole mt genome, with the highest number in *cox1* gene (*n* = 14). The intergenic regions have a total length of 29,350 bp, accounting for 18.63% of the whole genome length (**Table 1**). The whole A+T content of the mt genome was 69.8% (**Table 1**), consistent with the characteristic of AT-rich in the Hypocreales fungal mt genome, ranging from 69.0 to 73.5% (**Table 1**).

All PCGs began with a canonical start codon ATG or GTG (**Table 2**) and most terminated with the stop codon TAA, except *cox1, cox3*, and *rps3*. As revealed in many fungi, the ATG initiation codon of *nad5* followed immediately after the termination codon of *nad4L*, with an overlap of a base “A”; the genes of *nad2* and *nad3* were uninterrupted (Kouvelis et al., 2004; Shen et al., 2015). The codon frequency analysis showed that a total of 63 codons were used for transcription, with the absence of CGC (Supplementary Table S4). The six most frequently used codons (TTA, ATA, TTT, AAT, TAT, and AAA) reflect the biased usage of A/T nucleotides (Supplementary Table S4). The fraction of codons encoding the hydrophobic amino acids (Met, Trp, Phe, Val, Leu, Ile, Pro, Ala, accounting for 42.59%) (Supplementary Table S4) could explain the hydrophobic nature of respiratory membrane complexes.

A total of 27 tRNA genes were identified in the mt genome of *O. sinensis* coding for all 20 amino acids (**Table 3**), alleviating the need for tRNA import into the mitochondrion from the cytoplasm (Kolesnikova et al., 2000). The presence of tRNA-W recognizing the UGA codon indicates that the *O. sinensis* mt genome is translated according to genetic code 4 (Fox, 1987). As a unique characteristic of Hypocreales mtDNAs, most of the tRNA genes in *O. sinensis* were organized into three clusters with minor differences, except for five tRNAs scattered as a single gene across the mt genome (**Figure 1**). The single tRNA genes were suggested to play a role in transcription or recombination events (Saccone et al., 2002).

**Table 3:** Transfer RNA genes in the mt genome of *O. sinensis*.

tRNA	Codon usage	Start	End	Length(bp)	Strand	tRNA	Codon usage	Start	End	Length(bp)	Strand
tRNA-Thr(T)	ACA	19715	19786	72	+	tRNA-Cys(C)	UGC	79892	79963	72	+
tRNA-Glu(E)	GAA	20017	20089	73	+	tRNA-Arg(R)	AGA	111323	111393	71	+
tRNA-Met(M)	AUG	20093	20163	71	+	tRNA-Tyr(Y)	UAC	139615	139698	84	+
tRNA-Met(M)	AUG	20166	20238	73	+	tRNA-Asp(D)	GAC	139721	139793	73	+
tRNA-Leu(L)	UUA	20239	20321	83	+	tRNA-Ser(S)	AGC	140661	140741	81	+
tRNA-Phe(F)	UUC	23951	24023	73	+	tRNA-Asn(N)	AAC	142587	142658	72	+
tRNA-Lys(K)	AAA	24188	24260	73	+	tRNA-Ile(I)	AUA	146772	146843	72	+
tRNA-Ala(A)	GCA	25669	25741	73	+	tRNA-Gly(G)	GGA	148122	148192	71	+
tRNA-Gly(G)	GGA	26401	26471	71	+	tRNA-Val(V)	GUA	151005	151077	73	+
tRNA-Leu(L)	CUA	27782	27865	84	+	tRNA-Ile(I)	AUC	151708	151779	72	+
tRNA-Gln(E)	CAA	28238	28311	74	+	tRNA-Ser(S)	UCA	155672	155757	86	+
tRNA-His(H)	CAC	28500	28575	76	+	tRNA-Trp(W)	UGA	155865	155936	72	+
tRNA-Met(M)	AUG	29557	29627	71	+	tRNA-Pro(P)	CCA	157390	157462	73	+
tRNA-Arg(R)	CGU	50205	50275	71	+						

*“+” means located on the positive strand.*

### Phylogenetic Analysis of *O. sinensis*

**FIGURE 2:**
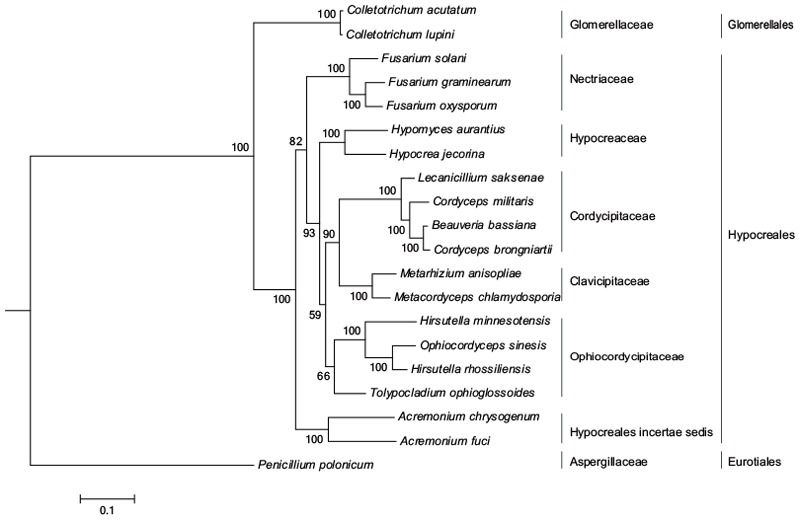
ML phylogeny of *O. sinensis* based on 14 PCGs in mt genome. The phylogenetic tree was inferred from a concatenated alignment of 14 PCGs (*atp6, atp8, atp9, nad1-nad6, nad4L, cob*, and *cox1*-*cox3*) using ML analysis. Numbers above branches specify bootstrap percentages (500 bootstrap replicates). MEGA 7.0 was used to determine the best evolutionary model (GTR+R) and RAxML 8.0 was used to infer the phylogenetic tree.

A ML phylogenetic tree of 20 taxa was inferred using 14 conserved PCGs associated with the OXPHOS system. *O. sinensis* was most closely related to *Hirsutella rhossiliensis*, with a bootstrap value of 100% (**Figure 2**), and these species then formed a sub-cluster with *H. minnesotensis* and *Tolypocladium ophioglossoides*. The cluster of these four fungi is consistent with the traditional classification that they are all in Ophiocordycipitaceae. As Lin et al. (2015) found, all entomopathogenic fungi formed a cluster in this ML phylogenetic tree, including the fungi in Clavicipitaceae (*Metarhizium* and *Metacordyceps*), Cordycipitaceae (*Cordyceps, Beauveria*, and *Lecanicillium*), and Ophiocordycipitaceae (*Hirsutella, Ophiocordyceps*, and *Tolypocladium*), while three *Fusarium* plant pathogens were clustered together. The topology of the ML tree agreed with that based on BI (**Supplementary Figure S1**), but differed slightly from the topology obtained using the NJ method (**Supplementary Figure S2**). In the NJ tree, *T. ophioglossoides* was clustered with *Metacordyceps chlamydosporia* and *Metarhizium anisopliae*, and the fungi in Cordycipitaceae branched earlier than that in the ML tree. The ML phylogenetic relationship inferred from the mt genome is principally consistent with the current Pezizomycotina taxonomic system (Taxonomy Database in NCBI).

### Introns

Notable in the *O. sinensis* mtDNA, it is the high degree of invasion by mobile DNA-elements (group I and group II introns). The number of introns exhibits remarkable variation in fungal mt genomes (Burger et al., 2003). The largest number of mt introns was documented for *O. sinensis* (*n* = 54) in Hypocreales in our analysis, while *M. chlamydosporia* has only one intron (**Table 1**) (Lin et al., 2015). In *O. sinensis*, 45 introns were found in 11 PCGs and 9 in 2 rRNA subunits, accounting for 84.17% (89,725 bp) and 15.83% (14,805 bp in *rnl*, 2025 bp in *rns*) of the whole intron length, respectively. As shown in other fungi, introns were abundant in *cox1, rnl*, and *cob* genes (Lang et al., 2007), containing 14, 8, and 6 introns, respectively, accounting for 94.82, 75.11, and 92.32% of each gene (**Table 2**). To these, *cox2*, and *nad5* should also be included since 93.18 and 81.15% of each gene were covered by introns, respectively. In contrast, several genes, such as *nad3, nad4*, and *atp8*, were intronless in *O. sinensis*.

BLASTx and RNAweasel results showed that 46 Group I introns and 6 Group II introns (Supplementary Table S3) were included in the *O. sinensis* mt genome, with two short introns failure to classify. Group I introns are reported to be dominant in fungal mt genomes, while group II introns are found more frequently in plant mt genome (Lang et al., 2007). Group I introns encode various HE genes with LAGLIDADG or GIY-YIG domain motifs, while group II introns mostly encode RTs (Lang et al., 2007). The motifs of HE genes and RTs in introns catalyze the transfer and site-specific integration of the introns into various genes (Lang et al., 2007).

### Open Reading Frames (ORFs)

In total, 73 ORFs were identified by MFannot in addition to the conserved genes (Supplementary Table S2), which is a little higher than that predicted by Li et al. (2015). The difference in ORF number may be due to different method used. The fungi in Hypocreales exhibited a broad spectrum of predicted ORFs in this study, from the least (*n* = 1) in *M. chlamydosporia* (Lin et al., 2015) to the most (*n* = 73) in *O. sinensis* (**Table 1**). The length of the ORFs in *O. sinensis* ranged from 306 to 2397 bp, with a total length of 59,421 bp accounting for 37.72% of the mt genome of *O. sinensis* (Supplementary Table S2). The variation in the number and length of predicted ORFs could partly explain the variation in genome size.

Among the 73 ORFs, 12 were free-standing, and the rest were located in introns (Supplementary Table S2). Among free-standing ORFs, only ORF_71 was predicted to be a DNA-directed RNA polymerase, while the functions of the others were unknown. In addition to the ORFs that exhibited similarities to GIY-YIG/LAGLIDADG endonuclease or RTs in introns, there were some ORFs encoding domains of intron encoded nuclease repeat and nuclease-associated module, which are possibly involved in DNA-binding (Supplementary Table S2).

### Molecular Evolution of *rps3*

The *rps3* gene was identified in the *rnl* intron of Hypocreales fungi, except *Acremonium fuci*. The *rps3* in *A. fuci* is freestanding. The length of *rps3* ranged from 1131 bp in *A. implicatum* to 1584 bp in *O. sinensis*. In *O. sinensis, rps3* was located in the IA intron of *rnl* (*rnl*_I8). A BLASTx search against the NCBI database showed that the *rps3* was fused to a novel LAGLIDADG HE gene. Half of *rnl*-I8 was homologous to *rps3* in *H. rhossiliensis* and half was similar to the HE gene from *Ophiostoma novo-ulmi* subsp. *americana* (Gibb and Hausner, 2005).

**FIGURE 3:**
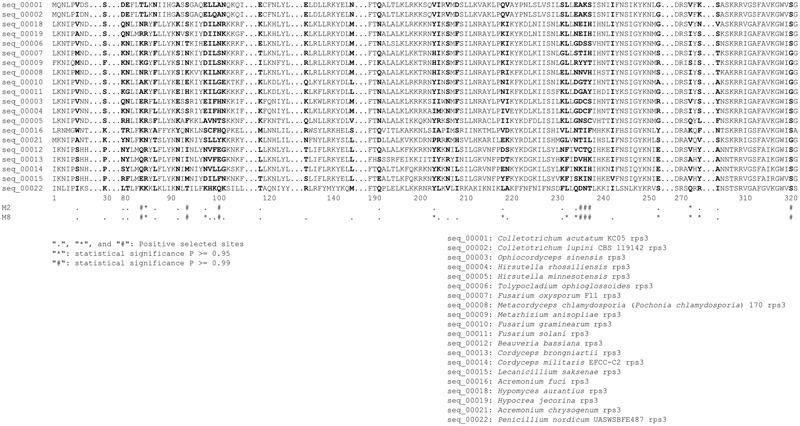
Positive selection sites across *rps3* in Hypocreales. Positive selection sites were identified by Bayes Empirical Bayes dN/dS values and labeled with symbols of “.”, “^∗^” (*P* ≥ 0.95) and “#” (*P* ≥ 0.99).

The phylogenetic relationships among the Hypocreales inferred from *rps3* genes were different from that based on 14 PCGs (**Supplementary Figure S3**). The sub-clusters (Hypocreaceae, Cordycipitaceae, Clavicipitaceae, and Ophiocordycipitaceae) in Hypocreales were similar between these two phylogenetic trees, except Nectriaceae. In the phylogenetic tree based on *rps3, Fusarium oxysporum*, belonging to Nectriaceae, was clustered with *M. anisopliae* rather than *Fusarium* spp. Moreover, the Cordycipitaceae cluster was separated from the Ophiocordycipitaceae cluster in the phylogenetic tree inferred from *rps3*. To measure the selective pressure of the *rps3* genes in Hypocreales, dN/dS values were calculated. The values were 0.2400, 0.1075, 0.1075, 0.1023, and 0.0848, when the adopted models were M0 (one-ratio), M1a (neutral), M2a (selection), M7 (beta), and M8 (β and ω), respectively. However, the LRT statistic for comparing M7 [lnL (log likelihood value) = -10,788.91] and M8 (lnL = -10,702.06) is 173.70 [2Δ = 2 ^∗^ (10,788.91–10,702.06) = 173.70], with a *P*-value < 0.01 using the Chi-square test (with df = 2). To further confirm whether *rps3* genes in Hypocreales are evolving under positive selection or not, the values of dN/dS were calculated in relation to the outgroup *P. nordicum*. The values of dN/dS were above 1.0 in Hypocreales (**Supplementary Figure S3**). Moreover, we explored a total of 36 sites in *rps3* with the values of dN/dS > 1 (**Table 4** and **Figure 3**), and 16 sites were statistically significant (*P* ≥ 0.95) (**Table 4** and **Figure 3**).

**Table 4:** Log-likelihood values and parameter estimates for *rps3* genes in Hypocreales.

Model	ℓ	Estimates of parameters	Positively selected sites
M1a (neutral)	-10810.63	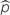 _0_ = 0.94987, 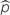 _1_ = 0.05013	Not allowed
M2a (selection)	-10810.63	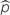 _0_ = 0.94987, 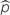 _1_ = 0.00563, 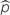 _2_ = 0.04450 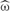 _0_ = 0.06045, 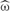 _1_ = 1.00000, 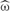 _2_ = 1.00000	6V, 80D, 81E, **84T**, **85L**, 87N, 92A, **94S**, 98E, **101A**, 119E, 192Q, 204V, 219Q, 232S, 235E, **236A**, **237K**, **238S**, 253G, **273V**, 275K, 305S, **321S**
M7 (beta)	-10788.91	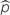 = 0.06958, 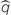 = 0.60258	Not allowed
M8 (β and ω)	-10702.06	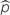 _0_ = 0.98400, 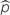 _1_ = 0.01600 *p* = 0.15774, 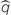 = 2.18092, 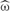 _s_ = 1.46813	6V, 30S, 80D, 81E, **84T**, **85L**, 87N, 92A, **94S**, **98E**, 99L, 100L, **101A**, 102N, 119E, 135E, 145N, 192Q, **204V**, 205I, 207V, 209D, **219Q**, 220V, 231L, **232S**, **235E**, **236A**, **237K**, **238S**, 242I, **253G**, **273V**, **275K**, 305S, **321S**

*p is the number of parameters in the ω distribution. Positive selection sites with *P* ≥ 95% were shown in bold.*

### Repetitive Sequences in the mt Genome of *O. sinensis*

Repetitive genes are considered as putative elements for recombination or regulation (Ghikas et al., 2006). A local self BLASTn of the *O. sinensis* mt genome against itself revealed 710 repetitive sequences (e-value < 10^-7^) with a total length of 77,583 bp, accounting for 49.25% of the whole mt genome (**Figure 1**). As shown in the mt genome of *Phlebia radiata* (Salavirta et al., 2014), the abundant repeat sequences were almost exclusively localized into intronic and intergenic region in *O. sinensis*, in particular between positions 110 to 30 kb (clockwise, **Figure 1**). Reputer identified a total of 3490 bp (2.22%) repeats in the *O. sinensis* mt genome, including 30 forward repeats (61–128 bp, in total 2446 bp), 13 palindromic repeats (61–90 bp, in total 968 bp), and 1 reverse repeat (76 bp) (**Table 5**). Tandem Repeats Finder found 44 tandem repeats (2–123 bp in copy size), with an average length of 63 bp, and accounting for 1.76% (2772 bp) of the mt genome (**Table 5**).

**Table 5:** Repetitive sequences in the mt genome of *O. sinensis.*

Type	Number	Repeat size (bp)	Total length (bp)	Percentage of genome (%)
Forward repeats	30	61–128	2446	1.55
Palindromic repeats	13	61–90	968	0.61
Reverse repeats	1	76	76	0.05
Tandem repeats	44	1–123	2772	1.76
Microsatellite DNA	43	1–6	729	0.46

Simple sequence repeats (also known as microsatellites) comprise tandemly repeated genetic loci of 1–6 bp (Tautz and Renz, 1984). SSRs were found in PCGs (*nad5, cox1*), rRNA (*rnl*), and non-coding regions (intronic and intergenic regions, Supplementary Table S5), with being more abundant in non-coding regions than in exons, as previously found in nuclear genome (Karaoglu et al., 2005). It assumed that SSRs might play an active role in genome evolution by creating and maintaining genetic variation (Tautz et al., 1986), and serve a functional role in gene expression regulation by influencing transcriptional activity or protein–protein interactions (Gerber et al., 1994; Kashi et al., 1997). Among these SSRs, most (36/43) were consisted of mono-nucleotide repeats, while the di-, tetra-, penta-, and poly-nucleotide repeats were found with much lower frequency (Supplementary Table S5). The reason may be that longer repeats have higher mutation rates and less stability (Wierdl et al., 1997). In agreement with the previous studies in fungi (Karaoglu et al., 2005), a majority of sequences rich in A/T were observed. All mono-nucleotide repeats were consisted of A/T repeats and the di-nucleotide repeats were AT/TA, with G/C only found in the less identified tetra-, penta-, and poly-nucleotide repeats.

### DNA Modification Analysis

**FIGURE 4:**
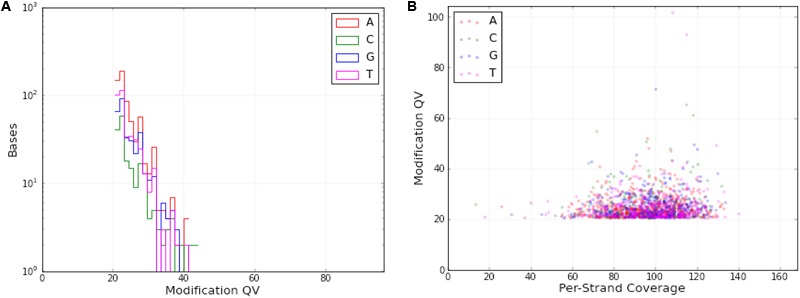
DNA modifications in the *O. sinensis* mt genome. **(A)** Distribution of the number of DNA modification bases with different modification quality value (QV). **(B)** Scatter plot of the DNA modifications with different per-strand coverage. At each genomic position, modification QV were computed as the –10 log (*P*-value) for representing a modified base position, based on the distributions of the kinetics of interpulse durations (IPD ratios) from all reads covering this position and from *in silico* kinetic reference values. Each dot represents a position on either strand with a modQV larger than 20. The color specified the nucleotide base, on which the modification was detected. Adenosines were colored in red, guanosines were colored in green, cytimidines were colored in blue, and thymines were colored in purple.

DNA modifications were determined in the mt genome of *O. sinensis* in parallel with the acquisition of primary sequence data by SMRT sequencing, based on analysis of the kinetics of DNA synthesis reactions. DNA modifications differ by various modification types, including 5-methylcytosine (5mC), 6-methyladenine (6mA), 4-methylcytosine (4mC) and 5-hydroxymethylcytosine (5-hmC), which can be identified by a series of methyltransferases at specific motifs. A genomic position is covered by several sequenced DNA fragments, and the modQV score shows the consistency by which a specific modification is observed. In the *O. sinensis* mt genome, a total of 1604 sites were determined with an average modQV score of 24.68 at an average coverage of approximately 96x (**Figure 4** and Supplementary Table S6). There were 783 modification sites located on the forward strand versus 821 located on the backward strand (**Figure 4** and Supplementary Table S6). The plot of modification scores against sequencing coverage displayed a dominant signal for modified adenosines and thymine bases (pink and burgundy dots in **Figure 4**). Twenty-eight 4mC (0.13%) and 10 6mA (0.017%) modification sites were identified in the *O. sinensis* mt genome (**Figure 1** and **Table 6**). The 6mA levels were lower than previously reported for nucleotide methylation (0.048–0.21%) in eukaryotic nuclear genomes (Mondo et al., 2017). Most 6mA and 4mC were distributed in intergenic regions (between tRNA and *nad4L*/*nad6/cox2*, or between tRNA and tRNA) or in intron regions of different genes (e.g., *nad1*-*2, cox1*-*2, cob, rnl*), with only three located in the genes. Two 4mC were methylated on the backward strand in the regions of *nad2* and *nad5* genes, and one 6mA was methylated in the *nad4L* region (**Table 6**). The hypermethylation in promoters and introns, and hypomethylation in exons, are also found in the nuclear genome of eukaryotes (Volpe, 2005). It is reported that methylation is strand-specific within the mt genome (Bellizzi et al., 2013). However, methyl modifications in the *O. sinensis* mt genome were observed on both strands, including 4mC and 6mA modifications (Supplementary Table S6 and **Table 6**).

**Table 6:** 6mA and 4mC in the mt genome of *O. sinensis.*

Type	Site	Location	modQV	Strand	Type	Site	Location	modQV	Strand
6mA	33641	*nad2*_intron2	30	+	4mC	92477	*cox1*_intron6	41	-
4mC	33651	*nad2*_intron2	25	+	4mC	99214	*cox1*_intron10	33	-
4mC	36698	*nad2*	25	-	4mC	103645	*cox1*_intron12	29	-
4mC	42050	*cox2*_intron3	27	-	6mA	106958	*cox1*_intron13	43	+
6mA	42687	*cox2*_intron3	34	-	4mC	107482	*cox1*_intron13	33	-
6mA	46658	*cox2*_intron5	37	+	4mC	108067	*cox1*_intron13	38	-
6mA	50156	tRNA_R_*nad4L*	27	-	4mC	112158	*nad1*_intron1	30	-
4mC	50187	tRNA_R_*nad4L*	39	-	6mA	122026	*nad1*_intron4	48	+
6mA	50384	*nad4L*	39	-	4mC	130958	*atp6*_intron2	32	-
4mC	62448	*nad5*	39	-	4mC	147647	tRNA_I_tRNA_G	50	+
6mA	65521	*cob*_intron1	52	+	4mC	151590	tRNA_V_tRNA_I	29	-
4mC	72293	*cob*_intron3	61	+	6mA	151635	tRNA_V_tRNA_I	25	+
4mC	72810	*cob*_intron3	42	-	4mC	6591	*rnl*_intron3	32	-
4mC	72821	*cob*_intron3	43	-	6mA	17260	*rnl*_intron8	30	+
4mC	77747	*cob*_intron6	43	+	4mC	17266	*rnl*_intron8	55	+
4mC	81629	*cox1*_intron1	34	-	4mC	20529	tRNA_L_tRNA_F	20	+
4mC	82628	*cox1*_intron1	65	+	4mC	21252	tRNA_L_tRNA_F	42	+
4mC	85113	*cox1*_intron3	23	-	4mC	23245	tRNA_L_tRNA_F	20	+
4mC	86263	*cox1*_intron3	35	-	4mC	26565	tRNA_G_tRNA_L	25	+

Because DNA methylation is diverse, widespread, and often deposited by a diverse set of methyltransferases at specific target sequences (motif), a comprehensive understanding of the distribution and diversity of methylation motifs will benefit comprehension of methylation function and evolutionary history of the mt genome. Eight modification motifs “AANNN^m4^CAGCANNANNNNA,” five of which were recognized by 4mC methyltransferases, were detected in the *O. sinensis* genome by SMRT sequencing with a mean QV of 44.4 at a mean coverage of 90.4x (Supplementary Table S6). Among the five methylation motifs, four were in the introns of PCGs (*cob, cox1*, and *rnl*), one was in the an intergenic region (tRNA_I_tRNA_G).

## Discussion

Single molecule, real-time sequencing, which was recently developed by Pacific Biosciences, can achieve extraordinarily long reads (up to 40 kb) with no GC bias, so that it is suitable for sequencing the genomes with low GC and high repeat content. Moreover, SMRT sequencing enables direct genome-wide detection of diverse base modifications by monitoring the kinetic variations of single bases. Thus, SMRT sequencing is convenient for sequencing the mt genome of *O. sinensis* and revealing the base modification pattern of the mt genome. It has long been debated whether modified bases exist in mt genomes.

Previously, *O. sinensis* has been called *Cordyceps sinensis* or *Cordyceps* sp. due to its morphology. However, the colony characteristics of *O. sinensis* cultures are significant different from other *Cordyceps* spp. Most *Cordyceps* species produce bright color and fleshy stromata, while the stromata of *O. sinensis* are often darkly pigmented, tough, fibrous to pliant, and have aperithecial apices, which are the characteristics of *Ophiocordyceps* (Sung et al., 2007). Based on these diagnostic characters and several loci sequence analyses (*nrSSU, nrLSU, tef1, rpb1, rpb2, tub*, and *atp6*), *C. sinensis* was classified into Ophiocordycipitaceae and renamed “*O. sinensis*” in 2007 (Sung et al., 2007). Our phylogenetic tree (**Figure 2**) based on the 14 PCGs confirms the phylogenetic position of *O. sinensis* in Ophiocordycipitaceae.

The mt genome intron number dynamics in the compared fungi (from 1 to 54) are most likely caused by the gain or loss of introns (Haugen et al., 2005; Cuenca et al., 2016). The introns are probably acquired from ancestors or gained through horizontal gene transfer (Haugen et al., 2005; Lang et al., 2007). HE domains in group I introns help to splice the transcribed intronic RNA, and are then removed from the transcribed pre-mRNA resulting in a contiguous RNA transcript in the process of gene transcription (Taylor and Stoddard, 2012). However, not all group I introns possess auto-catalytic splicing activity, especially for the mini-introns (e.g., *rnl_*I5 and *nad6_*I1), which lack the typical features (Schafer et al., 1991; Lang et al., 2007). It is proposed that the loss of intron splicing activity may be compensated by some other assistant proteins (Kreike et al., 1987; Lambowitz and Perlman, 1990; Lang et al., 2007). If the numerous HE motifs in the *O. sinensis* mtDNA are active, the HE domains might regulate the transcription of their target genes or modify their target genes, as seen in bacterial viruses and the animal-pathogenic *Cryptococcus* spp. (Ma et al., 2009; Stoddard, 2011). In addition, the existence of HEs within fungal mtDNA genes could promote intron mobility, genetic diversity and adaptive responses for mt genomes, when the allelic recombination events may be impossible or rare in mtDNA due to maternal inheritance (Barr et al., 2005; Basse, 2010).

Within the fungi, *rps3* is extremely diverse in location and organization: some are lost, some are free-standing, some are incorporated into group I intron, and others have been invaded by HEs (Sethuraman et al., 2009). Among the Ascomycete fungi, group I intron-encoded *rps3* seems to have a rather complex evolutionary history (Sethuraman et al., 2009). The phylogeny of *rps3* genes inferred in this study displays an evolutionary pattern that is different from that based on 14 conserved PCGs in Hypocrealean fungi, similar to the report of Lin et al. (2015). The *rps3* in *A. fuci* was a free standing gene and was grouped with the intron encoded *rps3*, implying that the *rps3* gene in *A. fuci* was somehow relocated and the *rnl* intron was lost. *rps3* is prone to insertion and deletion, is a refuge for HE genes, and is subject to recombination, making it problematic to resolve taxonomic relationships using *rps3* (Sethuraman et al., 2009).

To evaluate the balance between purifying selection, neutral evolution and positive selection acting on *rps3*, the dN/dS ratio, an indicator of evolutionary pressure on a gene, was examined in Hypocreales fungi. A dN/dS value < 1.0 in Hypocreales indicates that *rps3* has evolved under functional constraints. However, the significance of LRT showed a clear signal of positive selection in *rps3*. In addition, the dN/dS values that were > 1 in Hypocreales in relation to the outgroup *P. nordicum* and the 36 explored positive selection sites suggest that *rps3* is under positive selection in Hypocreales fungi, which is in accordance with the results of Lin et al. (2015). *rps3* is involved in DNA repair and potentially has endonuclease activities in *Schizosaccharomyces pombe* and nuclear versions of *rps3* in human and *Drosophila melanogaster* (Neu et al., 1998; Lyamouri et al., 2002; Jang et al., 2004). Except for these model organisms, no functional studies confirm that mtDNA-encoded *rps3* genes are actually functional (Kim et al., 2009). Further studies are needed to determine if the *rps3* genes in the mt genomic introns produce functional proteins and how these intron-encoded *rps3* genes are expressed.

Base modifications are indispensable parts of comprehending biological processes such as host-pathogen interactions, DNA damage and DNA repair (Trygve, 2010). Among base modifications, DNA methylation has been one of the most studied modifications. It has emerged as a significant phenotypic determinant for disease susceptibility and pathogenesis in eukaryotes, and involved in the Restriction- Modification system in prokaryotes (Bernstein et al., 2010; Ershova et al., 2015). However, whether mtDNA can be the site of epigenetic modifications has long been mired in controversy because the mt genome is multi-copy, lacks canonical CpG islands and histones (D’Aquila et al., 2017). SMRT sequencing is a more innovative and sensitive technology by comparing to the first and second generation DNA sequencing. Its advent makes the discovery of mitochondrial epigenetics, including various DNA modifications, more convenient. In this study, we revealed a mitogenome-wide epigenetic DNA modification pattern of *O. sinensis* by SMRT sequencing, resolving the issue of whether or not epigenetic modifications exist in the mt genome of fungi.

Among the DNA modifications in the mt genome of *O. sinensis*, several 6mA and 4mC methylations have been identified, which are always found in prokaryotes and have the function of protecting against restriction enzymes, regulating virulence and controlling DNA replication, repair, and expression (Ratel et al., 2006). Bisulfite sequencing has enabled genome-wide surveys of 5mC methylation (Masser et al., 2016), a well-established epigenomic mark in eukaryotes, but the historic absence of tools for studying 6mA and 4mC modifications has precluded more comprehensive studies of 6mA and 4mC methylation. Recently, Mondo et al. (2017) firstly analyzed 6mA in fungi by SMRT, and revealed its role as a gene-expression-associated epigenomic mark. In this study, SMRT sequencing revealed that not only 6mA but also 4mC were presented in the fungal mt genome. Nucleotide modifications are one of the most evolutionarily conserved properties of RNA, and the sites of modification are under strong selective pressure (Li and Mason, 2014). Mitochondria are largely thought to originate from endosymbiotic α-proteobacteria species (Gray, 2012). Thus, the 6mA methylations, which are often found in α-proteobacteria, in the fungal mitogenome might be remnants of the methylation system from α-proteobacteria. However, the function of these epigenetic modifications in the mt genome of *O. sinensis* remains unknown.

Mitochondria are crucial for responses to hypobaria, hypothermia, and hypoxia due to their central role in energy production and consumption (Chandel and Schumacker, 2000; Chitra and Boopathy, 2013). Cold temperature and low oxygen pressure are the two most remarkable characters of high-altitude environments, where *O. sinensis* is endemic (Li et al., 2011; Yu et al., 2011). Current associations between the mt genome and high-altitude adaption is focused on the mtDNA content and polymorphisms of mt genes, including NADH dehydrogenases, cytochrome c oxidases, ATP synthases and cytochrome b (Luo et al., 2013). However, no studies had been conducted on mt epigenetics. In this study, we found only three methylation affecting coding regions (in *nad2, nad4L*, and *nad5*), while the rest is spread between intergenic regions and introns (Supplementary Table S6). Complex I in the mitochondria, including NAD1-6, NAD4L, are involved in collecting electrons from various donors and passing them to coenzyme Q, which then passes the electrons to Complexes III (including COB) and IV (including COX1-3), and finally to the final electron acceptor O_2_ to complete the electron transport chain. At the end of this chain, Complex V (including ATP6, ATP8, and ATP9) catalyzes the reaction between ADP and inorganic phosphorus to form ATP (Saraste, 1999). The DNA methylations in eukaryotes are closely related with DNA transcription and translation (Volpe, 2005). Therefore, we supposed that the methylations across the whole *O. sinensis* mt genome, covering protein-coding regions, introns, and intergenic regions, might be closely related to the expression of the electron transport subunits (*nad1*-*2, nad4L, nad6* in Complex I, *cob* in Complex III, c*ox 1*-*2* Complex IV, and *atp6* in Complex V), and thereby modulate the enzymatic activity of OXPHOS and the mt respiratory rate, and then change the ability to capture O_2_ and produce energy. Moreover, methylation of mtDNA is supposed to play an important role in mitochondrial biogenesis by affecting the binding affinity of transcription factor A to mtDNA, impacting the relative activity of promoters (Van der Wijst and Rots, 2015). As the mentioned above, the methylation of mtDNA in *O. sinensis* might be a genetic feature for adaptation to the cold and low PO_2_ environment at high altitudes (Volpe, 2005; Luo et al., 2013).

## Conclusion

Single molecule, real-time sequencing was applied to characterize the *O. sinensis* mt genome in order to mitigate problems with assembly due to high AT and repeat content of the mt genome. The phylogenetic tree inferred from 14 PCGs supports the phylogenetic position of *O. sinensis* in Ophiocordycipitaceae. A total of 36 sequence sites were explored with the values of dN/dS > 1, suggesting that positive selection acts on *rps3* in Hypocreales fungi. Furthermore, we have analyzed the DNA modification pattern of the mitogenome directly. This is the first report of methylation in a fungal mitochondrion, and we propose that methylations in the *O. sinensis* mt genome might closely relate with the environmental responses for adapting to the cold and low PO_2_ environments at high altitude sites where *O. sinensis* is endemic.

## Author Contributions

DL conceived this study. XK analyzed the data and drafted the manuscript. LH participated in the data analysis and prepared figures. PS participated in the data analysis. RL sequenced and analyzed the mt genome. All authors have read and approved the final manuscript.

## Conflict of Interest Statement

The authors declare that the research was conducted in the absence of any commercial or financial relationships that could be construed as a potential conflict of interest.

## Footnotes

1 https://www.ncbi.nlm.nih.gov/genomes/GenomesGroup.cgi?opt=organelle&taxid=451864

2 http://megasun.bch.umontreal.ca/RNAweasel/

3 https://www.ncbi.nlm.nih.gov/genome/organelle/

4 http://www.ebi.ac.uk/interpro/

## Supplementary Material

FIGURE S1 Bayesian phylogeny of *O. sinensis* based on 14 PCGs in mt genome. MrBayes 3.2.6 was applied to construct the phylogenetic tree. Numbers at the nodes are Bayesian posterior probabilities.

FIGURE S2 Neighbor-joining phylogeny of *O. sinensis* based on 14 PCGs in mt genome. Numbers above branches specify bootstrap percentages (500 bootstraps). The evolutionary distances were computed using the Tajima-Nei method. MEGA 7.0 was used to construct the phylogenetic tree.

FIGURE S3 Evolutionary characteristics of *rps3* for 20 different taxa. Numbers above branches specify bootstrap percentages (500 bootstraps). The evolutionary history was inferred using the Neighbor-Joining method and the evolutionary distances were computed using the Tajima-Nei method. Evolutionary analyses were conducted in MEGA7.0. The right of the species names in the phylogenetic tree are the dN/dS values, which are calculated based on the comparative analysis with the *rps3* sequence of *P. nordicum*, an outgroup for the phylogenetic analysis.

## Abbreviations

BI: Bayesian inference
dN: non-synonymous substitution
dS: synonymous substitution
HE: homing endonuclease
ML: maximum likelihood
modQV: modification quality values
NJ: neighbor-joining
OXPHOS: oxidative phosphorylation
PCGs: protein-coding genes
RT: reverse transcriptase
SMRT: single molecule, real-time
SSRs: simple sequence repeats
