# Supplementary figures and images for: SMRT Sequencing Revealed Mitogenome Characteristics and Mitogenome-Wide DNA Modification Pattern in Ophiocordyceps sinensis
Source: Front Microbiol. 2017 Jul 27;8:1422. doi: 10.3389/fmicb.2017.01422 (PMC5529405; doi:10.3389/fmicb.2017.01422)

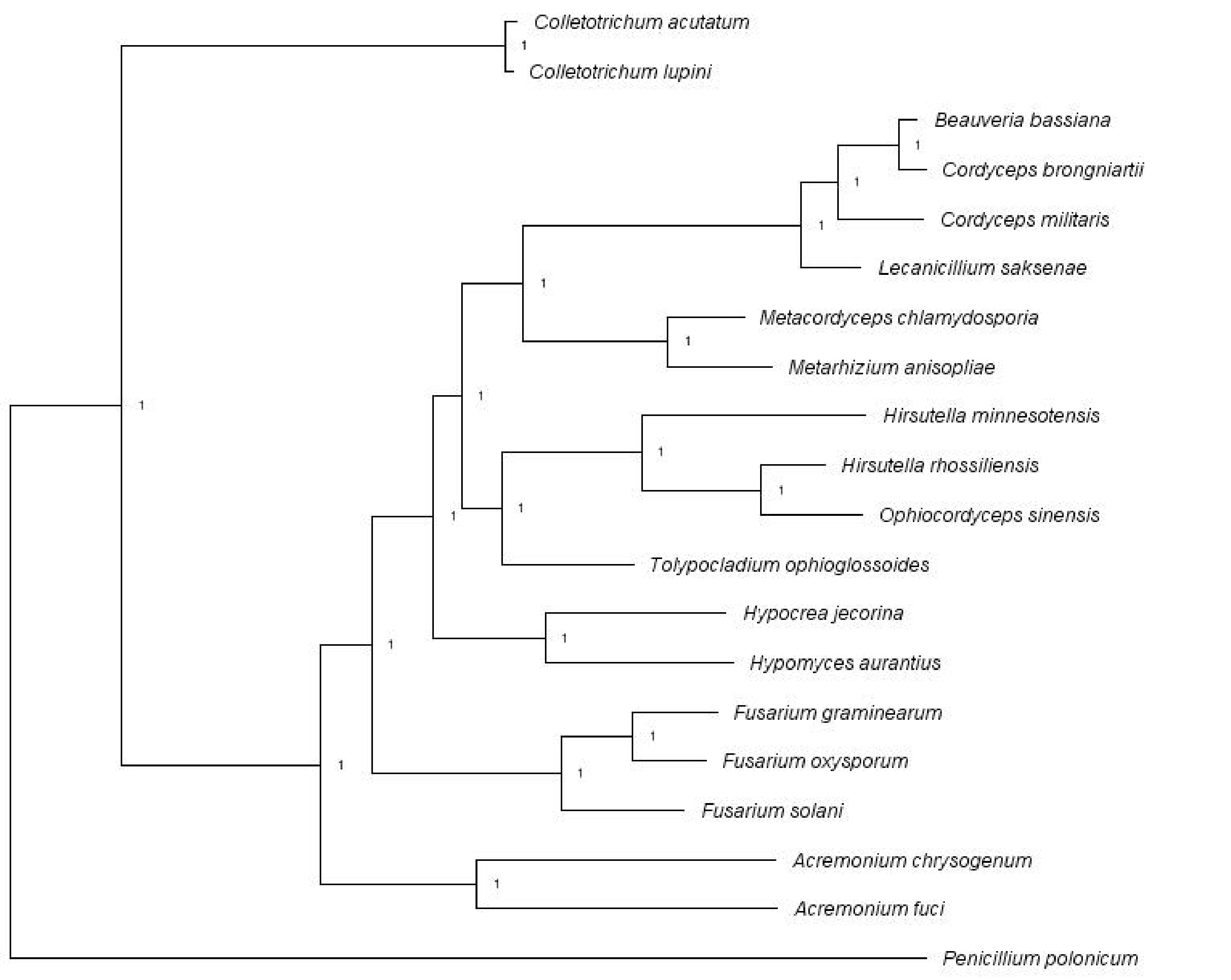

Supplement: FIGURE S1 — Bayesian phylogeny of O. sinensis based on 14 PCGs in mt genome. MrBayes 3.2.6 was applied to construct the phylogenetic tree. Numbers at the nodes are Bayesian posterior probabilities. [file Image_1.JPEG]

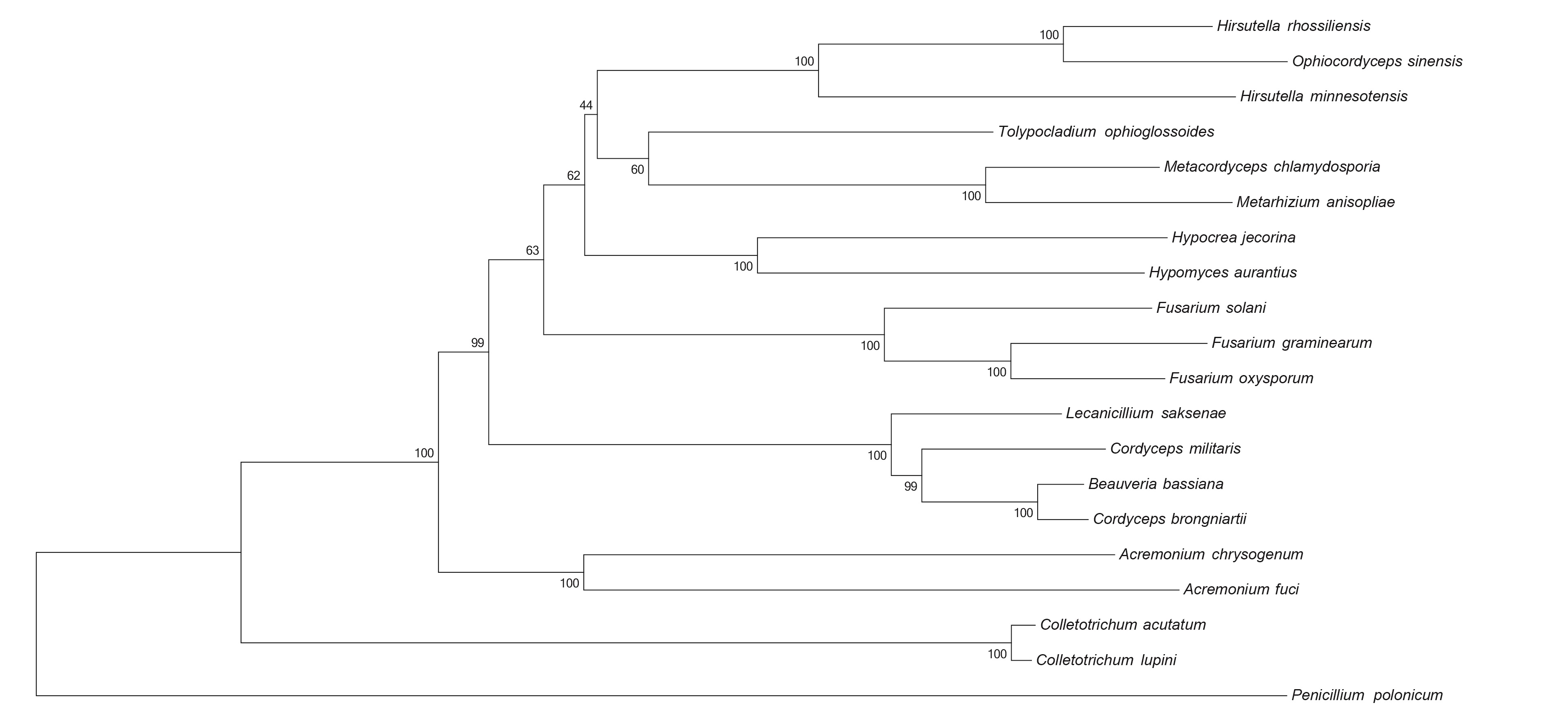

Supplement: FIGURE S2 — Neighbor-joining phylogeny of O. sinensis based on 14 PCGs in mt genome. Numbers above branches specify bootstrap percentages (500 bootstraps). The evolutionary distances were computed using the Tajima-Nei method. MEGA 7.0 was used to construct the phylogenetic tree. [file Image_2.JPEG]

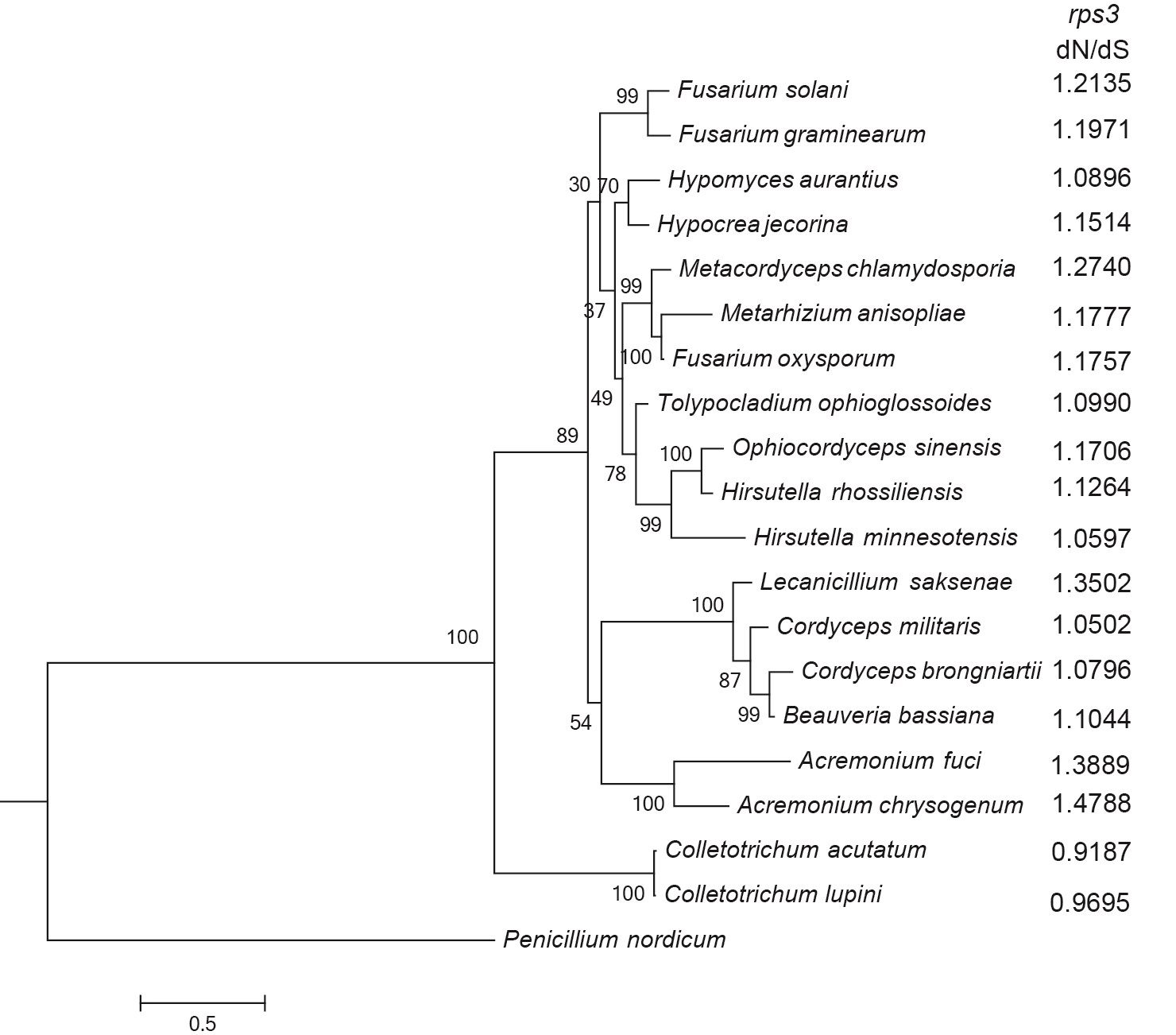

Supplement: FIGURE S3 — Evolutionary characteristics of rps3 for 20 different taxa. Numbers above branches specify bootstrap percentages (500 bootstraps). The evolutionary history was inferred using the Neighbor-Joining method and the evolutionary distances were computed using the Tajima-Nei method. Evolutionary analyses were conducted in MEGA7.0. The right of the species names in the phylogenetic tree are the dN/dS values, which are calculated based on the comparative analysis with the rps3 sequence of P. nordicum, an outgroup for the phylogenetic analysis. [file Image_3.JPEG]
